# Postural Orthostatic Tachycardia Syndrome in Spinal Cord Injury

**DOI:** 10.7759/cureus.41124

**Published:** 2023-06-29

**Authors:** Aditi Yadav, Raj Kumar Yadav, Osama Neyaz, Shabeeba Sherin P P, Anshini Gupta

**Affiliations:** 1 Department of Physical Medicine and Rehabilitation, All India Institute of Medical Sciences, Rishikesh, Dehradun, IND

**Keywords:** autonomic dysfunction, tachycardia, syncope, sci, spinal cord injury, pots, postural orthostatic tachycardia syndrome

## Abstract

Spinal cord injury (SCI), in addition to motor and sensory problems, may also lead to autonomic dysfunction. Postural orthostatic tachycardia syndrome (POTS) is one of them and has often been reported in traumatic brain injuries, multiple sclerosis, and other spinal cord pathologies. However, there is not much data on POTS in SCI even in extensive databases. We present a case of an adolescent female with paraplegia due to traumatic SCI. During her tilt table training, she started having episodes of sinus tachycardia associated with fatigue, dizziness, headache, palpitations, and presyncope with no orthostatic hypotension, after achieving 60 degrees of head tilt. After ruling out the common causes of tachycardia and syncope, a diagnosis of POTS was established. With pharmacologic and non-pharmacological measures, including metoprolol, increased fluid intake, and compression stockings, her symptoms resolved, and she was able to continue rehabilitation.

## Introduction

Spinal cord injury (SCI) disrupts the neuronal pathways, commonly leading to sensory and motor deficiencies. Autonomic dysfunction, another common consequence of SCI, results in a significant loss of perfect cardiovascular homeostasis of the body [1]. Autonomic dysreflexia, orthostatic hypotension (OH), and bradycardia are some common autonomic dysfunctions reported in SCI individuals [2]. People with SCI, particularly with neurological level T6 and above, frequently exhibit a decrease in peripheral and splanchnic circulation response to autonomic demands, thus reducing control of heart rate and cardiac output. The altered levels of plasma catecholamines, brought on by defective descending sympathetic regulation, also produce profound cardiovascular dysfunction. Autonomic dysfunction may also manifest as postural orthostatic tachycardia syndrome (POTS), causing inadequate cardiovascular regulation [3]. Although stress, injury, and surgery are some of the most common trigger factors for POTS, it has not been reported commonly in the literature in relation to SCI. This condition could severely affect a patient’s quality of life. Here the authors describe a case of POTS as a manifestation of autonomic dysfunction in traumatic SCI.

## Case presentation

**Figure 1:**
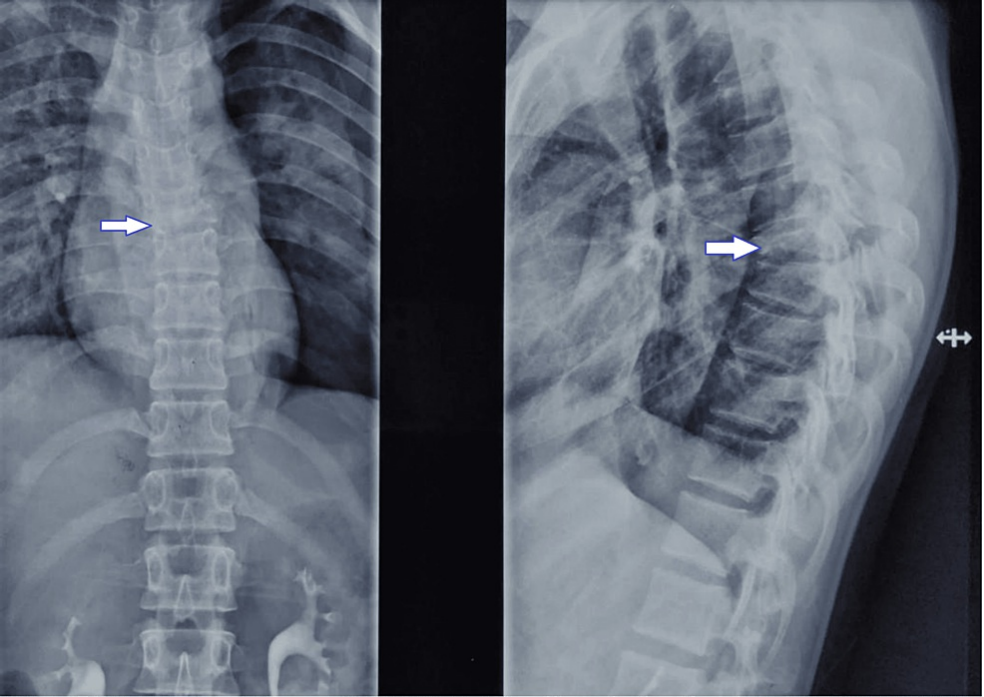
X-ray of vertebral injury – AP and lateral view AP, anteroposterior.

**Figure 2:**
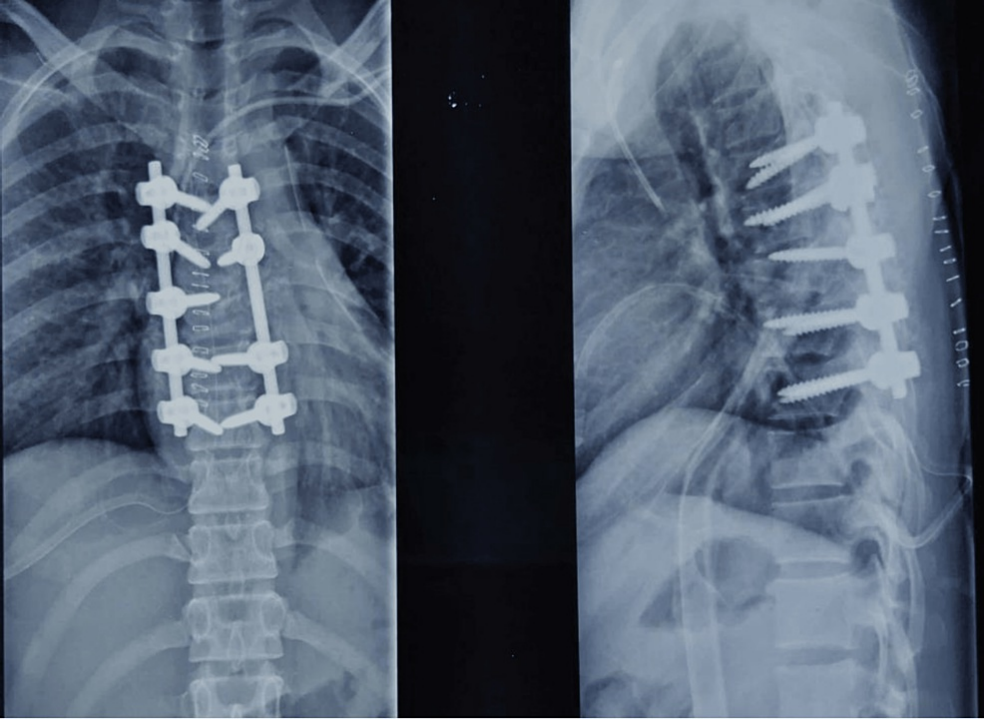
Postoperative X-ray after fixation – AP and lateral view AP, anteroposterior.

A female in her late teens had a fall from height, leading to a T7 vertebral burst fracture with spinal cord compression. Initially, she was managed surgically by posterior decompression and instrumentation from T4-T8 on the tenth day of the injury. After one month of injury, she presented in the Rehab ward as a traumatic paraplegia along with neurogenic bladder and bowel. Her examination findings were motor and sensory levels bilaterally at T7; neurological level of injury at T7; American Spinal Injury Association Impairment Scale (AIS)-B (Figures 1, 2).

In the Rehab ward, as an initial protocol of ambulatory training, she was put on a tilt table. After achieving 60 degrees of head tilt, her heart rate started increasing with symptoms of fatigue, dizziness, headache, palpitations, and presyncope. But her BP was maintained (Table 1).

**Table 1:** Head-up tilt data on the third day

	Degrees of tilt	Vitals
Third day	0 inclination	BP: 100/70 mmHg, HR: 100 bpm
	10 degrees	BP: 100/64 mmHg, HR: 100 bpm
	20 degrees	BP: 104/60 mmHg, HR: 108 bpm
	30 degrees	BP: 100/72 mmHg, HR: 120 bpm
	40 degrees	BP: 102/66 mmHg, HR: 126 bpm
	50 degrees	BP: 98/68 mmHg, HR: 135 bpm
	60 degrees - orthostatic symptoms appeared; stopped	BP: 110/68 mmHg, HR: 145 bpm
Duration of tilt was 1 hour (stopped if symptoms aggravated) at every session, inclination being increased at the rate of 10 degrees/10 minutes		

Her heart rate increased from a baseline of 100 bpm to 145 bpm at 60° inclination with associated symptoms of lightheadedness, uneasiness, and sweating. Her baseline blood pressure showed no change at 60° tilt. Repeated trials at the tilt table for three days showed similar symptoms with an increase in HR by 35-40 bpm at 60-70° inclination from baseline with no OH. A complete workup was done to find the cause of syncope with tachycardia but no hypotension.

The investigations done are mentioned in Table 2.

**Table 2:** Investigations WBC: White Blood Cell; HCT: Hematocrit; TSH: Thyroid-Stimulating Hormone; T3: Triiodothyronine; T4: Thyroxine; SGOT: Serum Glutamic Oxaloacetic Transaminase; SGPT: Serum Glutamic Pyruvic Transaminase; ALP: Alkaline Phosphatase; ECG: Echocardiogram; 2D ECHO: Two-Dimensional Echocardiography; ESR: Erythrocyte Sedimentation Rate; hsCRP: High-Sensitivity C-Reactive Protein.

Complete blood count		Reference values
Hemoglobin	10.8 g/dL	12-15 g/dL
WBC	5800/uL	4000-11000/uL
Platelet	2.39 lakhs/uL	1.5-4.0 lakhs/uL
HCT	33.8%	36-45%
Thyroid profile		
TSH	5.06 mcIU/mL	0.35-5.5 mcIU/mL
T3	1.21 ng/mL	0.89-1.76 ng/mL
T4	2.96 pg/mL	2.3-4.2 pg/mL
Liver function test		
SGOT	68 U/L	0-35 U/L
SGPT	37 U/L	0-35 U/L
Total bilirubin	0.56 mg/dL	0.3-1.2 mg/dL
Direct bilirubin	0.07 mg/dL	0-0.2 mg/dL
ALP	137 U/L	30-120 U/L
Kidney function test		
Blood urea	19.0 mg/dL	17-43 mg/dL
Serum creatinine	0.32 mg/dL	0.55-1.02 mg/dL
ECG	Sinus tachycardia	
2D ECHO study of heart	No structural abnormality	
Autonomic function test	Sympathetic and parasympathetic reactivity within normal range but parasympathetic dominance with heart rate response to lying to standing test decreased (Table 4 in appendices)	
ESR	23 mm/1st hour	0-20 mm/1st hour
hsCRP	3.00 mg/L	0-1 mg/mL
Serum vitamin B12	476 pg/mL	200-900 pg/mL
Serum ferritin	265 ng/mL	24-307 ng/mL

The differential diagnosis, in this case, was OH, vasovagal syncope (VVS), inappropriate sinus tachycardia (IST), neurally mediated syncope (NMS), and POTS. For a patient with SCI who has not been upright for over a month, due to impaired sympathetic activity and uninhibited parasympathetic activity in combination with cardiovascular deconditioning, the expected change in blood pressure would be a fall in systolic (SBP) and diastolic blood pressure (DBP) during the tilt-up, sufficient to meet the criterion for OH. The mean HR would be stable during the initial baseline period, increased during the tilt-up period by 15-20 bpm, and returned to near resting values on resumption of the supine position [4]. But in our patient, there was no fall in SBP or DBP, and HR increased by 35-40 bpm on head-up tilt. So OH was ruled out. VVS and NMS are common causes of frank syncope, but they result in bradycardia and hypotension in the upright posture. IST patients have elevated resting heart rates (>100 bpm) with no increase in standing.

Diagnostic criteria of POTS [5]

1) Heart rate increment >30 bpm within 5 minutes of standing or tilt-up.

2) Heart rate >120 bpm within 5 minutes of tilting up or standing.

3) Orthostatic symptoms consistently persist.

4) Absence of other overt causes of tachycardia.

**Figure 3:**
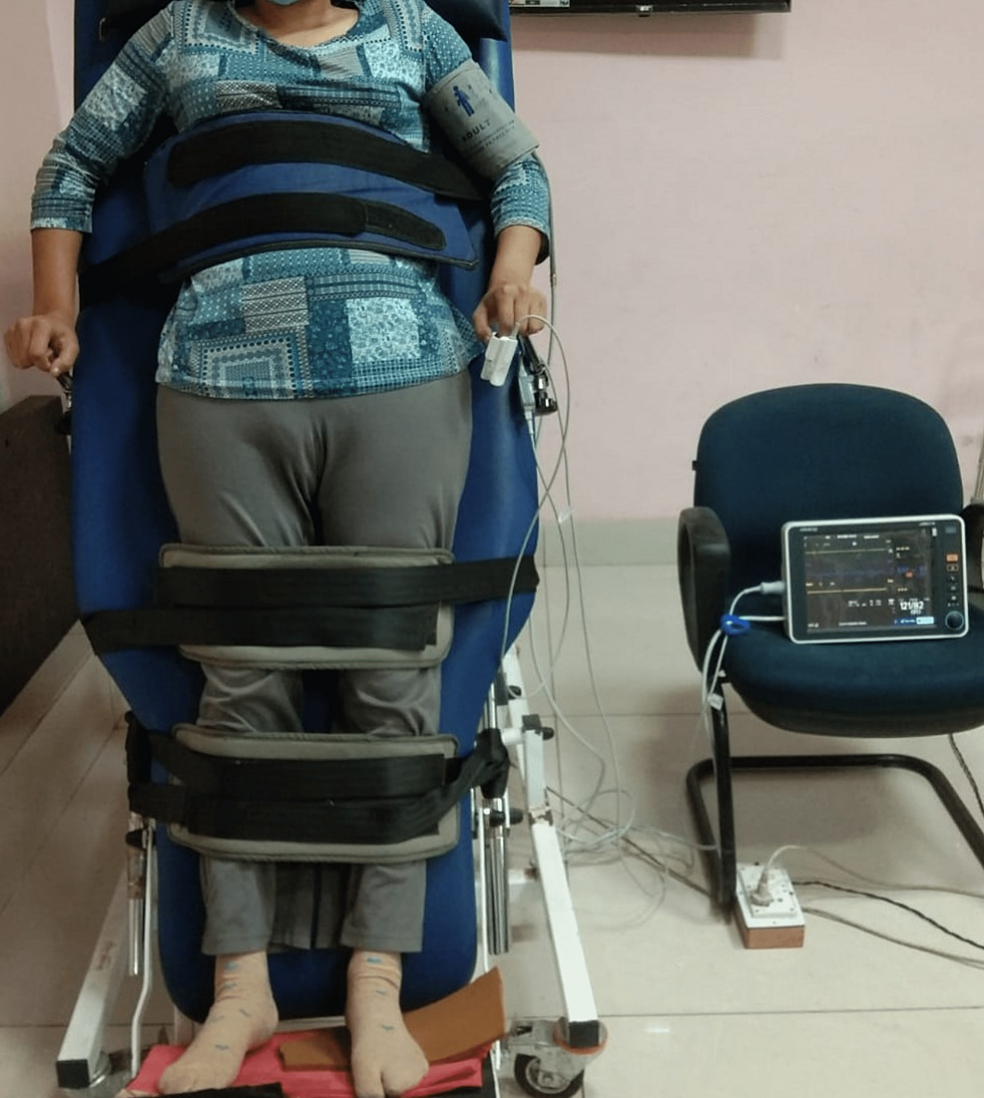
Patient on the tilt table

Blood investigations, ECG, and 2D echo testing were done to rule out various secondary causes of tachycardia, i.e. anemia, thyrotoxicosis, pheochromocytoma, arrhythmia disorder, infection, dyselectrolytemia, etc. No secondary attributable cause was ascertained for tachycardia. The patient underwent an autonomic function test on the Power Lab 8/35 machine with 8 inputs for recording external signals, which resulted in a normal study except for postural tachycardia >30 bpm during the tilt table test. Thus, a diagnosis of POTS was made (Figure 3).

After being diagnosed with POTS, she was started on the tablet metoprolol 12.5 mg twice daily. Her daily water intake was increased from 1.5 L to ∼3 L; the patient was asked to apply abdominal binders and compression stockings during the entire day, whenever she was off the bed. Within the next five days, the patient achieved 80 degrees on head tilt up and then was shifted to body-weight-assisted robotic gait training. After 10 days, she could stand in a parallel bar with gaiters and ankle foot orthosis (AFO) for 30 minutes. The rest of the structured rehabilitation protocol for improving ambulatory status, endurance, respiratory functions, and upper body strength was continued along with the tablet metoprolol 12.5 mg once daily. The patient's orthostatic symptoms were resolved within five days of starting her on tablet metoprolol 12.5 mg twice daily, increased fluid intake, and use of compression stockings. Within one week she achieved 80 degrees’ head tilt up with no increase in heart rate above 100 bpm. After achieving standing, the patient was discharged with a home-based exercise program and some home modification suggestions. At the time of discharge, her postural tachycardia had settled. Her functional independence score (FIM) was 48 at the transfer to the rehab ward and 82 at the time of discharge.

With respect to the patient's perspective, she was pretty satisfied at the time of discharge, as she finally succeeded in standing with calipers and managing her day-to-day activities independently, which was looking impossible during the initial days of rehabilitation when the symptoms of POTS appeared.

## Discussion

POTS is a common cause of syncope and presyncope, owing to dysfunction in the autonomic system. The search for similar cases via online databases like PubMed, Scopus, Cochrane Library, Embase, and ScienceDirect led us to some case reports of POTS in spinal cord pathologies, traumatic brain injury, PTSD, and pregnancy, but no article with POTS in SCI (see Table 3). Google Scholar was also utilized for open-access articles. The terms used to locate the case reports related to ours were Postural orthostatic tachycardia syndrome, POTS, spinal cord injury, SCI, trauma, traumatic brain injury, spinal cord pathology, concussion, and autonomic dysregulation.

**Table 3:** Case reports with POTS POTS: Postural Orthostatic Tachycardia Syndrome; PTSD: Post-Traumatic Stress Disorder; MRI: Magnetic Resonance Imaging; C: Cervical Vertebra; y: Years; M: Male; F: Female.

S. No.	Age/sex	Primary diagnosis	Abnormal symptoms	Method of diagnosis of POTS	Risk factors /triggers associated with POTS	Management of POTS	Reference
1	30y/F	Multiple sclerosis	Postural lightheadedness, subjective tremulousness	Head-up tilt table test was consistent with POTS. MRI was suggestive of demyelination at C2 and C4-C5 levels.	Female, reproductive age group, high spinal cord pathology	Metoprolol 25 mg twice a day, spironolactone 50 mg daily	Tripathi and Bernitsas[6]
2	42y/F	Chiari Type I malformation	Occipital headaches associated with nausea and vomiting, chronic fatigue, syncopal episodes, and palpitations in an upright position	Schellong test (active standing test for measuring blood pressure and heart rate changes under gravitational stress)	Female, reproductive age group, high spinal cord pathology	Surgical osteodural decompression with posterior fossa craniectomy and C1 laminectomy	Prilipko et al.[7]
3	37y/M	Traumatic brain injury	Resting tachycardia	Tilt table test, plasma norepinephrine level in an erect posture	Trauma, surgery	Increased fluid intake	Pande et al. [8]
4	19y/F	Type 1 diabetes, PTSD	Postural palpitations, lightheadedness	Autonomic function testing, norepinephrine levels with postural change	Female, reproductive age group, traumatic stress	Ivabradine, psychosocial therapy	Meyer et al.[9]
5	33y/F	Pregnancy	Recurrent blackouts, palpitations	Head-up tilt test consistent with a diagnosis of POTS	Female, pregnancy	Increased fluid intake, avoid triggers such as prolonged standing, graduated exercises.	Sidhu et al. [10]
6	50y/F	Cervical kyphosis and thoracic scoliosis	Neck pain, postural palpitations, lightheadedness	Head-up tilt test consistent with a diagnosis of POTS	Female, work-related anxiety and stress, cervical spondylosis	Spinal manipulation, thermal ultrasound therapy	Chu and Lin [11]

In the normal population, the prevalence of POTS has been found to be 0.2% [12]. In SCI, there is insufficient data regarding its prevalence as it is often missed as a diagnosis. POTS is a definite clinical entity characterized by sustained sinus tachycardia without hypotension and symptoms of syncope and presyncope when the person is made to stand or tilt. Also, these symptoms of orthostatic intolerance are found for a prolonged period, as seen in our case. The majority of POTS patients are between 13 and 50 years of age, with a female-to-male ratio of 4-5:1. These females are primarily of the reproductive age group. Also, approximately 13% of patients reportedly have an orthostatic intolerance history in the family [13]. Orthostatic symptoms can be pain or discomfort in the chest, palpitations, lightheadedness, blurring of vision, headache, breathlessness, fatigue, trembling, nausea, vomiting, or syncope; most of these were presented by our patient. There is often a history of acute stressors immediately before the beginning of symptoms like viral infections, pregnancy, or surgical and traumatic insults [14]. Sometimes, it can be a late complication of traumatic brain injury. In our case, the patient was a young female with a traumatic SCI managed surgically, having syncope and postural tachycardia with no hypotension, and all the secondary causes of tachycardia were negative.

POTS pathophysiology is less understood and is a combination or overlap of many proposed theories. POTS could result from mild autonomic neuropathy, hypovolemia and abnormal renin-aldosterone response, and genetic or acquired deficiency of norepinephrine receptors leading to a hyperadrenergic state [15]. Some patients with POTS have an increased association with autoimmune disorders, suggesting immune-mediated pathophysiology of the autonomic nervous system [16].

**Figure 4:**
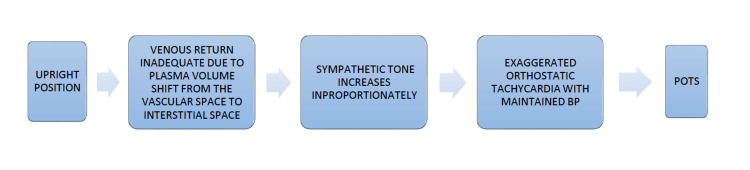
Pathophysiology of POTS POTS: Postural Orthostatic Tachycardia Syndrome. Illustrated by Dr. Raj Kumar Yadav.

Based on all the heterogenic etiologies, a common pathway could be derived (Figure 4).

Patient education is a crucial component of managing POTS cases. For the prevention of severe symptoms, the patient should be advised to avoid extremes of temperature, do strenuous exercise in upright posture in the initial phases, and be vigilant immediately in the post-prandial period after eating. Increasing water and salt intake are advisable. Medicines that aggravate sinus tachycardia and orthostatic tachycardia should be stopped immediately, if possible. Every POTS management plan should include exercise training. The exercise protocol starts with recumbent aerobic and resistance training progressing to upright exercises. These have reported benefits in reducing tachycardia and the symptoms, thus improving the quality of life [17].

In POTS, many drugs have been tried to improve orthostatic intolerance. Beta-adrenoreceptor antagonists like propranolol, metoprolol, and bisoprolol are the most often utilized medications for treating POTS, with an equal efficacy between propranolol and bisoprolol. Recent studies have shown that adding pyridostigmine to the beta-blockers does not have any significant benefit [18]. Ivabradine may help POTS patients by reducing their heart rates and improving their symptoms, according to a recent meta-analysis [19]. In a few studies, the oral alpha agonist prodrug midodrine has been proven to improve vascular regulation in POTS cases. Fludrocortisone has also been used in some studies [20].

In this particular case, we used metoprolol, and the results were satisfactory.

## Conclusions

POTS is a common autonomic dysfunction causing symptoms of syncope and presyncope. Though not very common, it can be found in SCI. With prompt suspicion and appropriate investigations, more common autonomic dysfunctions in SCI should be excluded. And, once the diagnosis of POTS is confirmed, appropriate beta-blockers like metoprolol along with non-pharmacological measures can be used to manage it effectively.

## Figures and Tables

**Figure 5:**
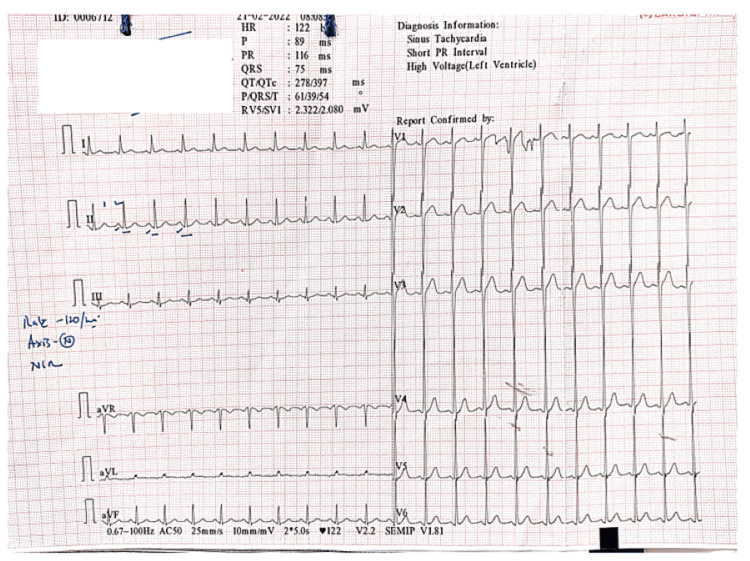
ECG of the patient at rest

## Appendices

**Table 4:** Autonomic function test LF/HF: Low Frequency/High Frequency; SDRR: Standard Deviations of All the R-R Intervals; pRR50: Percentage of Normal Successive RR Intervals That Differ by More Than 50 milliseconds; BP: Blood Pressure; E:I ratio: Expiratory:Inspiratory Ratio.

Name of the test	Results	Normal range
Heart rate variability	SDRR value is normal	
	pRR50 is normal, suggestive of normal parasympathetic tone	
	LF/HF ratio is less than normal suggestive of parasympathetic dominance	LH/HF ratio: 1.2
Postural tests:		
Head-up tilt		
1) Change in systolic BP	Fall of 4 mmHg	Fall of <10 mmHg
2) 30:15 ratio	0.88	>1.04
Deep breathing tests:		
1) Change in heart rate	31.00	>15 bpm
2) E:I ratio	1.51	>1.21
Valsalva manoeuver test: Valsalva ratio	2.30	>1.21
Isometric handgrip test: Rise in diastolic BP	13	>10 mmHg
Cold pressor test: Rise in diastolic BP	10	>10 mmHg
